# Core subjects at the end of primary school: identifying and explaining relative strengths of children with specific language impairment (SLI)

**DOI:** 10.1111/1460-6984.12137

**Published:** 2014-12-03

**Authors:** Kevin Durkin, Pearl L H Mok, Gina Conti-Ramsden

**Affiliations:** † School of Psychological Sciences and Health, University of StrathclydeGlasgow, UK; ‡ School of Psychological Sciences, The University of ManchesterManchester, UK; § School of Psychological Sciences, The University of ManchesterManchester, UK

**Keywords:** specific language impairment, English studies, mathematics education, science education, Key Stage 2, procedural learning

## Abstract

**Background:**

In general, children with specific language impairment (SLI) tend to fall behind their typically developing (TD) peers in educational attainment. Less is known about how children with SLI fare in particular areas of the curriculum and what predicts their levels of performance.

**Aims:**

To compare the distributions of performance of children with SLI in three core school subjects (English, Mathematics and Science); to test the possibility that performance would vary across the core subjects; and to examine the extent to which language impairment predicts performance.

**Methods & Procedures:**

This study was conducted in England and reports historical data on educational attainments. Teacher assessment and test scores of 176 eleven-year-old children with SLI were examined in the three core subjects and compared with known national norms. Possible predictors of performance were measured, including language ability at ages 7 and 11, educational placement type, and performance IQ.

**Outcomes & Results:**

Children with SLI, compared with national norms, were found to be at a disadvantage in core school subjects. Nevertheless, some children attained the levels expected of TD peers. Performance was poorest in English; relative strengths were indicated in Science and, to a lesser extent, in Mathematics. Language skills were significant predictors of performance in all three core subjects. PIQ was the strongest predictor for Mathematics. For Science, both early language skills at 7 years and PIQ made significant contributions.

**Conclusions & Implications:**

Language impacts on the school performance of children with SLI, but differentially across subjects. English for these children is the most challenging of the core subjects, reflecting the high levels of language demand it incurs. Science is an area of relative strength and mathematics appears to be intermediate, arguably because some tasks in these subjects can be performed with less reliance on verbal processing. Many children with SLI do have the potential to reach or exceed educational targets that are set at national levels for TD children.

**What this paper adds?**

**What is already known on the subject?**

It is established that, as a group, children with SLI tend to fall behind their TD peers in educational attainment. Less is known about how children with SLI fare in particular subject areas and what predicts their levels of performance.

**What this study adds?**

The study confirms that children with SLI do perform, overall, below national norms in core subjects at the end of primary school but shows also that some attain the levels expected of TD peers. Performance varies among subjects (poorest in English, with relative strengths in Science). Children with SLI undoubtedly face many hurdles due to the language of education but may profit from learning opportunities that draw on other capacities, such as visual representation.

## Introduction

In general, children with specific language impairment (SLI) tend to fall behind their typically developing (TD) peers in educational attainment, but there are two important qualifications to this generalization. The first is that there are some exceptions. For example, some individuals with SLI do well in school and progress through to higher education (Dockrell *et al*. 2007, Durkin *et al*. 2009). The second is that recent studies indicate overall improvements in the educational attainments of children with SLI compared with a couple of decades earlier (Conti-Ramsden *et al*. 2009, Dockrell *et al*. 2007, Durkin *et al*. 2009, Johnson *et al*. 2010). These are important qualifications because they indicate that expectation levels should not be set low for children with SLI and that good-quality education can support these children towards optimal outcomes.

In this context, it is essential to learn more about how children with SLI fare in particular areas of the curriculum and what predicts their levels of performance. Although it is clear that language impairment makes learning processes more difficult, we lack information on the extent to which it bears on outcomes in different school subjects. In England, where the present study was conducted, all children follow a government-directed curriculum, labelled Key Stage 2, from 7 to 11 years of age. Children are assessed both by their teachers and by national tests at the end of this period, which marks the end of primary schooling. Until 2011, children were assessed on three core subjects: English, Mathematics and Science. Since the independent review led by Lord Bew (Bew 2011), there are no longer formal national assessments in Science at Key Stage 2. In this study, we report historical data prior to the Bew review; hence, we are able to comment on children's performance in Science as well as English and Mathematics. For each subject, two sources of information may be obtained for a given child (teacher, test) and extensive normative, national data are available against which to examine children's attainment levels. Key Stage 2 scores correlate highly with scores in subsequent secondary school examinations, e.g. General Certificate of Secondary Education (GCSE) at age 16 (Strand 2006). Previous research with the present sample of children with SLI confirms that they do perform poorly, on average, compared with national norms (Conti-Ramsden *et al*. 2002). However, to date, predictors of outcomes have not been systematically tested for children who have been assessed in all three core areas: English, Mathematics and Science at 11 years. We consider first reasons why language impairment should bear on each subject area.

### English

The Key Stage 2 assessments and tests in English are designed to measure children's performance in reading, writing, grammar, vocabulary, punctuation and spelling. All these are difficult tasks for children with SLI. Language difficulties predict literacy difficulties in preschool as well as school-age children (Bishop and Adams 1992, Botting *et al*. 2006, Harlaar *et al*. 2008, Justice *et al*. 2013). This is a consistent finding across standardized as well as less formal measures of language and literacy. Children with SLI tend to be poorer than TD peers on measures of story comprehension and recall (Bishop and Adams 1992, Norbury and Bishop 2002) and, in narrative productions, they show deficits in topic maintenance, event sequencing and conveying implicit relations (Mäkinen *et al*. 2014, Miranda *et al*. 1998). Hence, assessment in English is addressing some of the principal difficulties experienced by children with SLI and could be expected to be a challenging area for them.

On the other hand, children with language difficulties do receive some help and therapy, so it is possible that targeted interventions may be supportive of their general development in English language skills. To address this possibility, we base our observations on children who were attending language units at the beginning of Key Stage 2 (7 years). We take into account the type of educational placement a child received later in the period, comparing those who by age 11 years enter mainstream education without additional language support, those who enter the mainstream but do receive language support, those who attend specialist language schools or language units and those who attend other special schools or units.

### Mathematics

The Key Stage 2 assessments and tests in mathematics are designed to measure children's understanding of the number system (integers, fractions and decimals), simple algebraic ideas, shape, space and measures, and handling and drawing inferences from data. As with most areas of the curriculum, teaching in mathematics is highly dependent on language. The curriculum is delivered in talk and texts; much of the language of school mathematics is specialized, sometimes using esoteric terminology and sometimes using everyday words in unfamiliar ways (Durkin and Shire 1991, Ellerton and Clarkson 1996, Ginsburg 2009). Developments in language skills are clearly associated with developments in numerical and mathematical abilities (Negen and Sarnecka 2012). Children with language impairments are at risk of arriving at school with already relatively poor numerical skills. Nelson *et al*. (2011) reported that 4-year-olds with severe language delay scored 1 SD below norms on a maths test measuring counting skills and simple addition and subtraction.

Previous research with children with SLI indicates that they do lag behind typical peers in school progress in number and Mathematics (Arvedson 2002, Cowan *et al*. 2005, Donlan *et al*. 2007, Durkin *et al*. 2013, Fazio 1999, Kleemans *et al*. 2011, 2012), though this depends to some extent on which abilities are tested. For example, Arvedson (2002) and Donlan *et al*. (2007) found, in preschoolers and primary age children, respectively, that on some number tasks designed to minimize verbal processing, children with SLI performed as well as age-matched TD peers. This raises the possibility that while Mathematics education may well be difficult for children with SLI, some aspects of working with numbers and abstract symbols may be less taxing. Thus, if language impairment does have a general impact on education, then we would predict that children with SLI will fall behind their TD peers. Nevertheless, because some mathematical work is less demanding of language processing, the level of performance in this subject area may be superior to that in English (which, by its very nature, is highly language-oriented). Mathematics may be a relative area of strength for these children.

### Science

The Key Stage 2 assessments and tests in Science cover a broad range of topics, including general principles of scientific enquiry (planning, obtaining and evaluating evidence), life processes (in humans and non-humans), materials and their properties, and physical processes (such as electricity, force, motion, light and sound). To engage with the relevant conceptual and procedural phenomena once again involves extensive use of language (Lemke 1990, Mercer *et al*. 2004, Tippett 2009). The Key Stage 2 Science curriculum requires children not only to describe but also to explain, and this makes higher order demands of language ability (Matson and Cline 2012). Wellington and Osborne (2001: 2) observe that ‘Every Science lesson is a language lesson’. They argue that learning the language of Science is a major part of Science education, and that language is one of the major hurdles facing all children learning Science.

Despite the importance of language in Science education, and despite the evident burden this could place on children with SLI, very little empirical research has been addressed to how these children perform in this area. However, Matson and Cline (2012) provide preliminary evidence to indicate that some of the visual scaffolding provided in some Key Stage 2 Science education may be helpful to children with language impairment. In a study of 7–11-year-olds, they found that, compared with TD peers, children with SLI had more difficulty in scientific tasks that required expressive language. However, there were no differences between the groups on tasks requiring receptive language when scaffolding was provided (in the form of picture cards on a magnetic board). The study was exploratory, with a small sample size, but it does suggest that some areas of Science education might be amenable, even helpful, to children with SLI because of lesser dependence on verbal processing. As with Mathematics, then, we would expect a general disadvantage to children with SLI in Science education compared with TD peers but, because Science may sometimes draw on other modes of representation and action, it is possible that performance in this subject would be superior to performance in English and a relative strength for these children.

In sum, there were three main purposes to the present investigation. First, for a large sample of children with SLI, we aimed to compare the distributions of performance in the three core Key Stage 2 subjects: English, Mathematics and Science (measured in teacher assessments and national tests) with the known national distributions; previous research led to the expectation that children with SLI would have less successful outcomes than the national norms. The second purpose was to test the possibility that performance would vary across the core subjects, such that the area most extensively dependent on language ability, namely English, would be the area of greatest difficulty for children with SLI and correspondingly that, relative to English, performance would be superior in Mathematics and/or Science. We also considered the possibility that this effect might be moderated by the kind of educational placement the child had experienced. The third purpose was to examine the extent to which language impairment predicts performance in the core subjects. We predicted that severity of language impairment would bear on attainment, such that the more severe the language difficulty, the poorer the educational outcome. We tested this prediction by examining both concurrent language (at age 11) and early language (at age 7) skills and by assessing the contribution of language skills once nonverbal IQ was taken into account.

## Methods

### Participants

The participants were originally part of a wider study: the Manchester Language Study (Conti-Ramsden and Botting 1999, Conti-Ramsden *et al*. 1997). The original cohort of 242 children, which consisted of 186 boys (76.9%) and 56 girls (23.1%), was recruited from 118 language units attached to English mainstream schools, and represented a random sample of 50% of all Year 2 children (approximately 7 years of age) attending language units for at least half the school week. Children were excluded if they were reported by their teachers as having frank neurological difficulties, a diagnosis of autism, hearing impairment or a general learning disability. At age 11, when the children were in their final year of primary school (Year 6), a total of 200 of the original 242 children were able to participate. There were no significant differences in expressive language, receptive language or PIQ between those children participating at 11 years and the 42 children who did not take part in this stage of the study.

For the purpose of this study, children were included if they had teacher assessments and/or test results in all three Key Stage 2 core subjects (English, Mathematics and Science). This resulted in 176 participants, of whom 132 (75%) were boys. All children had English as a first language. A small number of children, 22 (12.5%), had exposure to languages other than English at home. At 11 years, 100 children (56.8%) were attending mainstream schools, with the vast majority (72%) receiving support of some kind within this setting. For the remaining 76 children, 52 (29.6%) were in specialist language units or language schools, and 24 (13.6%) were attending other special schools or units such as those specializing in moderate learning difficulties. Of the 118 participants for whom maternal education data were available, 28% had no formal education qualifications, 44.1% had mothers educated up to GCSE/‘O’-level standard, a further 19.5% up to ‘A’-level or equivalent and 8.5% had a further education qualification (university or college degree or equivalent).

Profiles of the participants at ages 7 and 11 are shown in table1. Note that different assessments were used for ages 7 and 11.

**Table 1:** Profiles of participants at ages 7 and 11

	Age 7 years			Age 11 years		
	*N*	Mean	SD	*N*	Mean	SD
Age months (year; month)	176	85.3 (7;1)	3.9	176	131.0 (10;11)	5.1
Receptive language raw scores^a^	171	10.3	3.4	176	15.3	2.9
Receptive language standard scores^a^	171	84.4	11.2	176	87.2	15.2
Expressive language raw scores^b^	171	22.0	7.5	176	46.6	14.0
Expressive language standard scores^b^	171	83.6	10.2	176	74.4	11.8
Receptive and expressive language composite scores^c^	171	32.3	10.0	176	61.9	16.0
						
PIQ standard scores^d^	170	106.2	14.8	175	86.7	23.8

Notes:

a Test for Reception of Grammar (TROG).

b Bus Story Test at age 7; CELF-R Recalling Sentences at age 11.

c Sum of TROG and Bus Story Test raw scores at age 7; sum of TROG and CELF-R Recalling Sentences raw scores at age 11.

d Raven's Coloured Progressive Matrices at age 7; WISC-III Block Design and Picture Completion at age 11.

The average standard scores for receptive language at both ages and for expressive language at age 7 were all around 1 SD below the normal population mean, while expressive language at age 11 was more than 1.5 SD below. The mean performance IQ (PIQ) scores fell from 7 to 11 years. At age 7, they were slightly above the population mean and at age 11 years just within 1 SD below. The use of different assessment tools may partially account for the drop in PIQ. However, instrument changes do not fully account for the data. The research findings using a scaling procedure that accounts for instrument changes has demonstrated that approximately one third of children with SLI experience a drop in PIQ from childhood to adolescence (Conti-Ramsden *et al*. 2012).

### Measures

#### National Curriculum Key Stage 2 Assessments

National Curriculum Key Stage 2 teacher assessment and test results from 1999 for the three core subjects: English, Mathematics and Science, were obtained from teachers for each child, using questionnaire format and were followed up by telephone interview where necessary (Conti-Ramsden *et al*. 2002). The teacher assessment for English is the overall average of attainment levels in speaking and listening, reading, and writing, while the English test result is derived from averaging separate reading and writing tests scores. For the other two subjects, Mathematics and Science, there is a single attainment level based on teacher assessment and a single attainment level based on test results.

For teacher assessment, the levels of attainment are represented by 1–6, with level 1 indicating the lowest level and level 6 the highest. Pupils working towards level 1 are assigned the level ‘W’. For test results, level 2 represents the lowest level that can be achieved and level 6 the highest. Although these outcomes are strictly ordinal in nature (e.g. pupils cannot achieve level 3.75 or 4.62 in the national curriculum), we will be treating the outcomes as continuous variables. This is possible, in this case, given that there are a reasonable range of categories (levels 1–6), with a fair spread of observations and an approximately normal distribution. Children who are entered for the test are considered to be able to cope with level 2 materials. However, children whose performance is not forthcoming are not awarded a numerical level but instead are assigned the level ‘N’. Those children who are considered to be working below the level assessed by the test, i.e. level 2, are not entered and they are assigned the level ‘B’. Children can also be disapplied from teacher assessments or the tests usually because of special educational needs. Children can also be absent on the day particular tests are given. Cases where children were disapplied or absent have been excluded from this study.

### Early test battery at 7 years

#### Receptive and expressive language

Receptive language at age 7 was assessed using the Test for Reception of Grammar (TROG; Bishop 1982). This is a test of oral comprehension of syntax in which children are shown four pictures while the examiner reads a sentence. The child is asked to pick the picture that illustrates the sentence. These items begin very simply and progress to more complex grammatical sentences (e.g., ‘the cat the cow chases is black’). Items are organized into blocks of four grammatically related sentences. The number of blocks passed is noted to give the TROG raw score, which can then be transformed into a standard score. Expressive language at age 7 was assessed using the Bus Story Test (Renfrew 1991), which is part of the Renfrew Language Scales. In this assessment, the examiner tells the child a short story about a bus while the child looks through a book of pictures illustrating the story. The child must then retell the story as accurately as possible using the pictures as cues. Stories are audiotaped, transcribed and scored for the amount of correct information given. Two points are given for information central to the story, and one point for peripheral details, and these are summed to give the ‘total information score’, which can subsequently be converted into a standard score. The information score of the Bus Story was used because it has been shown to be a significant predictor of overall prognosis in children with SLI (Botting *et al*. 2001). The receptive and expressive language raw scores were highly correlated, *r* = 0.60, *p* < 0.001. For the purpose of this study, therefore, a composite score representing both the receptive and expressive language ability of the child at age 7 was derived by summing the TROG raw score and the Bus Story ‘total information score’.

#### Performance IQ

Raven's Coloured Progressive Matrices (Raven 1986) was used to assess participant's PIQ at age 7. This test presents the child with a series of patterns from which a ‘piece’ is missing, and the child is asked to choose from six alternative pieces the one that completes the pattern. The test is split into three sets of 12 patterns each and the number of correct answers is summed. This total score is then compared with age-relevant population norms.

### Concurrent test battery at 11 years

#### Receptive and expressive language

Receptive language at age 11 was also assessed by TROG (Bishop 1982). Expressive language at age 11 was assessed using the Recalling Sentences subset of the Clinical Evaluation of Language Fundamentals—Revised (CELF-R; Semel *et al*. 1987). This subtest is designed to assess recall and reproduction of surface structure as a function of syntactic complexity, and the child is required to repeat sentences of increasing complexity given verbally by the tester.

Following the same procedure used at age 7, a composite score representing the language ability of the child at age 11 is derived from summing the TROG and CELF-R recalling sentences raw scores.

#### Performance IQ

Performance IQ was assessed using the Block Design and Picture Completion subtests of the Wechsler Intelligence Scale for Children (WISC-III; Wechsler 1992). The raw score from each subtest is first converted to a *t*-score. The two are then summed and transformed to a standard score for use in this study.

### Procedure

Ethical approval was obtained from The University of Manchester and written informed consent was gained from all participants’ families. Children were visited at school and assessed individually in a quiet room or area with only the participant and a trained researcher present. The battery of psychometric tests was completed as part of the wider study. In nearly all cases, testing was completed in 1 day at the child's pace and with normal school breaks. Because of the large number of measures used, the numbers of data points available may vary from measure to measure. All statistical analyses were conducted using Stata/SE 12.0 (StataCorp 2011) and a two-tailed significance level of *p* = 0.05 was used.

## Results

### National Curriculum Key Stage 2 teacher assessment and test results

Tables2a and b show the grades awarded for Key Stage 2 teacher assessments and test results respectively, for the three core subjects of English, Mathematics and Science.

**Table 2a:** Key Stage 2 teacher assessment results in 1999 by academic subjects

Award code^a^	English (%)		Mathematics (%)		Science (%)	
	SLI sample	All schools	SLI sample	All schools	SLI sample	All schools
	(*N* = 168)	in England[Table-fn tf2-2]	(*N* = 168)	in England[Table-fn tf2-2]	(*N* = 168)	in England[Table-fn tf2-2]
W	1	0	2	0	1	0
1	9	1	9	1	4	1
2	37	6	33	5	27	3
3	42	25	36	24	43	20
4	10	48	16	48	22	53
5	1	19	4	22	2	23
6	0	0	0	0	0	0
Level 4 or above	11	68	20	69	24	75
Percentage at level 4 or above—all special schools in England in 1999	–	3	–	3	–	5

Notes: All percentages are rounded to follow practice by the Department of Education (DfE).

a Number codes represent the level of attainment where the child has been assessed by their teacher, with level 1 representing the lowest level and level 6 the highest; W, working towards level 1.

b All school types include maintained, independent and special schools (Department for Education (DfE) 2000).

Of the 176 participants, teacher assessment results were available for 168 (95.5%) children in all three subjects, and 112 (63.6%) had results for all three tests. Given our interest in comparing children's performance across school subjects, only children for whom results were available across all three subjects have been included in our analyses. For comparison, the percentages of pupils achieving each grade level in England in the year this study sample took the examinations, i.e. 1999 (Department for Education (DfE) 2000), are also presented. The reduced number of children with test results in our study was largely due to the exclusion of pupils who were disapplied from the test: 54 for English, 38 for Mathematics and 26 for Science. As mentioned above, these children, as well as those who were absent from the test, were excluded from our analyses. The correlations between teacher assessments and test results were strong for all three subjects (English, *r* = 0.81, *p* < 0.001; Mathematics, *r* = 0.84, *p* < 0.001; and Science, *r* = 0.75, *p* < 0.001).

### Performance in relation to national norms

The minimum level expected to be achieved by a majority of TD students at the end of Key Stage 2 is level 4 (though Stobart 2009 points out that the official expectation of 85% or above at this level is an aspiration rather than the reality). Participants were grouped depending on whether or not they had achieved this expected level (table3).

**Table 3:** Percentage of participants achieving level 4 or above in Key Stage 2 by type of placement at 11 years

	Teacher assessment (*N* = 168)			Test result (*N* = 112)		
	English (%)	Mathematics (%)	Science (%)	English (%)	Mathematics (%)	Science (%)
Mainstream school—without support	28	56	56	37	56	63
Mainstream school—with support	14	21	28	18	38	54
Language school or unit	4	9	15	25	20	30
Other special school or unit	0	0	0	0	0	0
All types of placement	11	20	24	22	36	47

Of the three Key Stage 2 subjects, English showed the lowest level of achievement, with only 11% and 22% of children reaching level 4 or above in teacher assessment and test results, respectively. These figures were considerably below the 68% and 71% for all schools in England, but were better than the 3% for both teacher assessments and test results for special schools. Performance in Science was the highest outcome, with 24% of participants achieving a minimum of level 4 in teacher assessment and 47% reaching the same level in test results. These percentages were again below the national averages of 75% and 78%, respectively, but considerably higher than the 5% and 10% reported for special schools. A substantial number of participants—120 for teacher assessment (71.4% of those with results available across all three subjects) and 54 for test results (48.2% of those with results available across all three subjects)—did not reach level 4 in any of the three subjects. Eighteen participants (10.7%) achieved level 4 or above in all three subjects in teacher assessment and 20 children (17.9%) reached this same level in all three test results. Note that none of the children attending other special schools or units achieved results at level 4 or above.

### Performance across the three core subjects

Performance across the three core subjects was examined, taking into account also placement type. Because different numbers of children were available with teacher assessment and test scores, we report on each of these modes of assessment separately.

#### Teacher assessment

**Figure 1:**
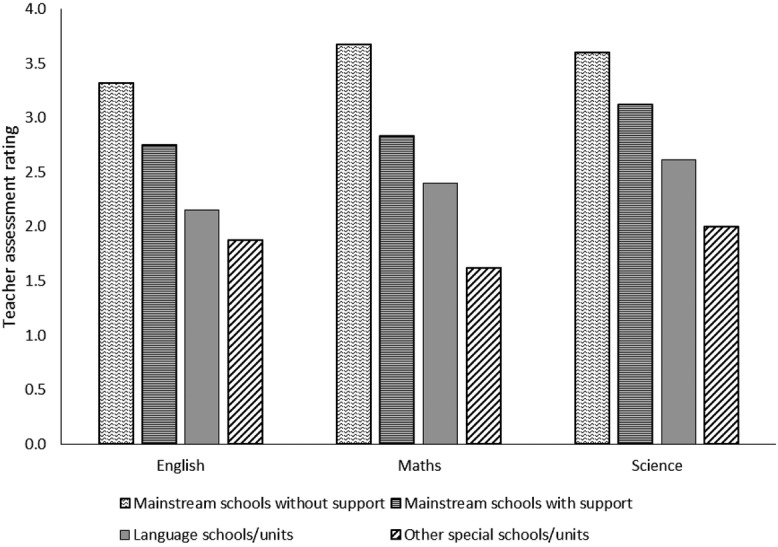
Teacher assessment ratings by placement type at age 11 years

Analyses of teacher assessment when the participants were aged 11 years were submitted to a 3 (Subject: English, Mathematics, Science) by 4 (Placement type: mainstream school without language support, mainstream school with language support, language school or unit, other special school/ unit) analysis of variance. For this analysis, teacher assessments were treated as continuous variables. Thus, the level ‘w’, i.e. those who were working towards level 1 (table2a), was recoded as ‘0’. The scale for teacher assessment thus spanned from 0 to 6, with 6 representing the highest level of attainment. This analysis yielded a significant main effect of subject, *F*(2, 328) = 14.56, *p* < 0.001, partial η^2^ = 0.08, and a significant main effect of placement, *F*(3, 164) = 30.58, *p* < 0.001, partial η^2^ = 0.63. These effects were qualified by a significant interaction between subject and placement, *F*(6, 328) = 2.58, *p* = 0.019, partial η^2^ = 0.05. This interaction is illustrated in figure 1.

To interpret this interaction, comparisons across subjects were examined for each of the four placement types separately. Analyses of simple effects were significant within each placement type. Pairwise contrasts, with Bonferroni adjustments, revealed the following pattern:

- Among children attending mainstream schools, not receiving additional language support, Mathematics assessments were superior to English assessments (*p* = 0.01).
- Among children attending mainstream schools and receiving additional language support, Science assessments were superior to Mathematics assessments (*p* = 0.001) and to English assessments (*p* < 0.001).
- Among children attending language schools or units, Science assessments were superior to English assessments (*p* < 0. 001).
- Among children attending other special schools or units, Science assessments were superior to Mathematics assessments (*p* = 0.03).
- (Pairwise comparisons not listed above did not yield significant differences.) Broadly speaking, then, assessments in English tended to be the lowest, and those in Science tended to be the highest (English = 2.5 (SD = 0.9); Mathematics = 2.7 (SD = 1.0); Science = 2.9 (SD = 0.9).

#### Test scores

Test results were submitted to the same 3 × 4 analysis of variance (ANOVA) design as above. Children who were working below the level assessed by the test and not entered for the test, and those who were entered but with no level awarded, i.e. those who were coded as ‘b’ and ‘n’, respectively (table2b), were recoded as ‘1’. Test results thus spanned from 1 to 6, with 6 representing the highest level of attainment. This analysis revealed a main effect of subject, *F*(2, 216) = 13.31, *p* < 0.001, partial η^2^ = 0.11, and a main effect of placement type, *F*(3, 108) = 10.2, *p* < 0.001, partial η^2^ = 0.45. The interaction between subject and placement type was not significant, *F*(6, 216) = 1.38, *p* = 0.23, partial η^2^ = 0.04.

**Table 2b:** Key Stage 2 test results in 1999 by academic subjects

Award code^a^	English (%)		Mathematics (%)		Science (%)	
	SLI sample	All schools	SLI sample	All schools	SLI sample	All schools
	(*N* = 112)^c^	in England^b^	(*N* = 112)	in England^b^	(*N* = 112)	in England^b^
B	3	3	3	3	3	2
N	14	2	16	2	4	1
2	11	1	11	1	4	0
3	50	20	35	23	41	16
4	18	48	30	45	38	51
5	4	22	5	24	9	27
6	0	0	0	0	0	0
Level 4 or above	22	71	36	69	47	78
Percentage at level 4 or above—all special schools in England in 1999	–	3	–	4	–	10

Notes:

a Number codes represent the level of attainment where the test has been taken, with level 2 representing the lowest level and level 6 the highest; B, working below the level assessed by test (not entered for test); N, no level awarded (entered).

b All school types include maintained, independent and special schools (Department for Education (DfE) 2000).

c For the SLI sample, percentages were calculated using the sum of pupils with codes B-6 as the denominator, i.e. excluding those who were absent or disapplied from the test. For all schools in England (DfE, 2000), the percentage of pupils for each grade reported was based on a denominator including those who were absent or disapplied, which consisted of about 2% of all pupils in England. There was insufficient information available that would allow for these percentages to be recalculated in the same way as for the SLI sample.

With regard to the main effect of subject, the mean scores were 2.8 (SD = 1.1) for English, 2.9 (SD = 1.2) for Mathematics, and 3.4 (SD = 1.0) for Science. Post-hoc analyses using Bonferroni corrections for multiple comparisons revealed that the mean test score for Science was significantly higher than those for English and Mathematics, *p*s < 0.001, but the difference between scores for the two latter subjects was not significant.

With regard to the main effect of placement type, post hoc comparisons with Bonferroni correction revealed that children from mainstream schools without support performed significantly better than those from mainstream schools with support, *p* < 0.001 (3.5 (SD = 1.0) and 3.1 (SD = 1.0) respectively), who in turn performed significantly better than those in language schools and units, *p* = 0.017 (2.9 (SD = 1.1). In addition, children from all these three placement types scored better than those from other special schools and units, *ps* < 0.001 (1.7 (SD = 0.9).

It is important to note that the psycholinguistic profiles of children attending different placements also differed (table4).

**Table 4:** Language profiles at 11 by placement

	Mainstream	Mainstream	Language	Other special
	without support	with support	school/unit	school/unit
Receptive standard scores	98.9 (15.7)	87.9 (13.0)	83.4 (14.7)	80.2 (14.5)
Expressive standard scores	84.2 (14.1)	74.2 (11.0)	70.3 (9.2)	72.2 (10.7)
Receptive and expressive composite	73.5 (13.6)	64.2 (12.4)	54.4 (16.9)	57.7 (17.0)
PIQ standard scores	99.4 (22.1)	87.5 (23.1)	85.4 (24.6)	72.3 (17.7)

Notes

Values are the mean (SD).

There was an overall significant difference in language abilities (receptive and expressive composites) across children in different placement types, *F*(3, 172) = 11.56, *p* < 0.001, partial η^2^ = 0.17. Post-hoc comparisons using Bonferroni adjustment for multiple comparisons revealed that children in mainstream schools with no support scored significantly better than those in mainstream with support, or those in language units or other special units (*ps* < 0.001–0.03). Similarly, examination of children's PIQ revealed overall significant differences by placement type, *F*(3, 171) = 6.17, *p* < 0.001, partial η^2^ = 0.10. Children in mainstream schools with or without support had higher PIQ than children in other special units, *p* = 0.032 and *p* < 0.001, respectively.

### Early and concurrent predictors of Key Stage 2 teacher assessments and test results

Linear regression analyses were conducted to examine possible predictors of Key Stage 2 teacher assessment and test results. Models were built in two steps: placement type (with mainstream school without support as the reference category) and PIQ standard scores at 11 were entered first, followed by the age 11 and age 7 receptive and expressive language composite score. The results are presented in table5.

**Table 5:** Linear regression analyses predicting Key Stage 2 results

	*b*	SE	β	*R*^2^	Adjusted *R*^2^
*Teacher assessment*					
English (*N* = 162)					
Step 1				0.341	0.324
Mainstream with support	–0.49**	0.17	–		
Language schools/units	–1.06**	0.18	–		
Other special schools/units	–1.22**	0.21	–		
PIQ at 11	0.01**	0.00	0.24		
Step 2				0.471	0.450
Mainstream with support	–0.32*	0.16	–		
Language schools/units	–0.67**	0.18	–		
Other special schools/units	–0.86**	0.20	–		
PIQ at 11	0.00	0.00	0.11		
Language composite at 11	0.01**	0.00	0.25		
Language composite at 7	0.02**	0.01	0.24		
**Mathematics (N = 162)**					
Step 1				0.412	0.398
Mainstream with support	–0.71**	0.19	–		
Language schools/units	–1.09**	0.20	–		
Other special schools/units	–1.71**	0.24	–		
PIQ at 11	0.01**	0.00	0.31		
Step 2				0.498	0.479
Mainstream with support	–0.55**	0.18	–		
Language schools/units	–0.71**	0.20	–		
Other special schools/units	–1.34**	0.24	–		
PIQ at 11	0.01**	0.00	0.20		
Language composite at 11	0.01*	0.00	0.14		
Language composite at 7	0.03**	0.01	0.25		
**Science (N = 162)**					
Step 1				0.391	0.376
Mainstream with support	–0.35*	0.17	–		
Language schools/units	–0.87**	0.18	–		
Other special schools/units	–1.30**	0.21	–		
PIQ at 11	0.01**	0.00	0.32		
Step 2				0.478	0.458
Mainstream with support	–0.22	0.16	–		
Language schools/units	–0.55**	0.18	–		
Other special schools/units	–0.97**	0.21	–		
PIQ at 11	0.01**	0.00	0.21		
Language composite at 11	0.00	0.00	0.07		
Language composite at 7	0.03**	0.01	0.31		
***Test results***					
**English (N = 109)**					
Step 1				0.229	0.200
Mainstream with support	–0.28	0.23	–		
Language schools/units	–0.53	0.29	–		
Other special schools/units	–1.19**	0.39	–		
PIQ at 11	0.01**	0.00	0.32		
Step 2				0.321	0.281
Mainstream with support	–0.13	0.22	–		
Language schools/units	–0.10	0.29	–		
Other special schools/units	–0.96*	0.39	–		
PIQ at 11	0.01	0.00	0.19		
Language composite at 11	0.02*	0.01	0.26		
Language composite at 7	0.02	0.01	0.14		
**Mathematics (N = 109)**					
Step 1				0.313	0.286
Mainstream with support	–0.25	0.24	–		
Language schools/units	–0.80**	0.30	–		
Other special schools/units	–1.52**	0.40	–		
PIQ at 11	0.02**	0.00	0.37		
Step 2				0.367	0.330
Mainstream with support	–0.11	0.24	–		
Language schools/units	–0.44	0.31	–		
Other special schools/units	–1.29**	0.41	–		
PIQ at 11	0.01**	0.00	0.27		
Language composite at 11	0.02	0.01	0.18		
Language composite at 7	0.02	0.01	0.13		
**Science (N = 109)**					
Step 1				0.305	0.279
Mainstream with support	0.08	0.20	–		
Language schools/units	–0.47	0.25	–		
Other special schools/units	–1.25**	0.33	–		
PIQ at 11	0.01**	0.00	0.33		
Step 2				0.353	0.314
Mainstream with support	0.19	0.20	–		
Language schools/units	–0.22	0.26	–		
Other special schools/units	–0.97**	0.34	–		
PIQ at 11	0.01**	0.00	0.26		
Language composite at 11	0.00	0.01	0.05		
Language composite at 7	0.02*	0.01	0.22		

Notes: For placement type, the reference category is mainstream school without support.

** *p* < 0.01;

* *p* < 0.05.

Because the same participants were included in the analyses of all three subjects when examining teacher assessments and test results separately, the relative importance of PIQ and language as concurrent predictors could be directly compared across the three subjects using the standardized regression coefficients.

For teacher assessments, language skills, in particular early language skills at 7 years, were significant predictors of performance in all three core subjects at the end of primary schooling. Comparisons of the standardized regression coefficients for all three subjects suggest that early language skills are a stronger predictor than PIQ for outcomes measured by teacher assessment.

For test results, language skills (at 11 years) featured as the strongest predictor for English and PIQ as the strongest predictor for Mathematics. For Science, both early language skills at 7 years and PIQ made significant contributions.

Results of Wald tests, conducted to examine the overall effect of the placement variable, showed that placement type was significant in all final models for teacher assessments, *ps* < 0.001, and for test results in Mathematics and Science, *ps* ≤ 0.010. The overall effect of placement type on the English test results after controlling for language abilities and PIQ, however, was not significant, *F*(3, 102) = 2.22, *p* = 0.091. In general, the predictors examined (placement type, PIQ and language abilities) were able to explain a larger proportion of the variance in teacher assessments (45–48%) than in test results (28–33%).

## Discussion

The first purpose in this study was to compare Key Stage 2 results for children with SLI with the known national distributions for the core subjects of English, Mathematics and Science. As anticipated, as a group, these children performed markedly less well than national norms. The minimum level expected to be achieved is level 4; between 68 and 78% of TD children achieve this level. As anticipated, the subject in which children with SLI did least well was English, where the percentages attaining the minimum level 4 were 11% in teacher assessments and 22% in test results. On this criterion, performance in Mathematics was intermediate (20% and 36%, respectively) and performance was best in Science (24% and 47%, respectively). Thus, children with SLI are clearly at a disadvantage in the core school subjects, and for some this is pervasive. It is useful to note here why children appear to perform better in tests rather than teacher assessments. Recall that a reduced number of children were allowed to take the formal tests. Children who are not deemed to be able to cope with the examinations are excluded. In this study, the tests were disapplied for a substantial number of children: 52 children in English, 38 children in Mathematics and 26 children in Science. Nevertheless, it is important to observe that in both Mathematics and Science, regardless of whether we examine teacher assessments or test results, some children appear able to manage or overcome disadvantages associated with language impairment to achieve at least the minimum age-appropriate levels.

The second purpose was to examine more closely differences in performance as a function of school subject and the educational placement type that the children had received. With respect to teacher assessments, we found that children tended to be evaluated more favourably in Science and, to a lesser extent, Mathematics, than in English. With respect to test results, Science scores were significantly superior to those obtained in English and Mathematics, which did not differ from each other. Overall, then, children with SLI tended to do best in Science, poorest in English, with intermediate results in Mathematics.

Educational placement effects showed that children attending mainstream schools without additional language support tended to fare best overall, followed by children attending mainstream schools where they did receive language support, then children attending a specialist language school or unit, and finally children attending other types of special school or unit. It is important to stress that the differences across placement types should not be interpreted as indicating differential effectiveness of the respective teaching environments. Children are likely to be allocated to different placements based on the severity of their language difficulties and other aspects of school performance. Hence, for example, children with milder language impairment are most likely to attend mainstream schools and they may be judged not to require additional language support; correspondingly, children with the most severe impairments are more likely to be placed in specialist schools or units. Examination of the psycholinguistic profiles of children attending different placements supports this interpretation.

The third purpose was to examine the extent to which language impairment predicts performance in the core subjects. We examined both longitudinal and concurrent relationships. Early language at age 7 years was a significant predictor of the results for teacher assessments in all three subjects: better early language at 7 years was associated with better performance in Key Stage 2. For test results, comparisons across the three subjects suggested that concurrent language at age 11 years was a stronger predictor for outcomes in English only. In contrast, language ability was not a significant predictor of results in Mathematics tests but PIQ was. For Science, both PIQ and language were significant predictors. This is consistent with the assumptions that English is a highly verbal domain and therefore particularly challenging to children with language impairments and that, while Mathematics and Science also draw on verbal abilities, there may be some aspects to the curricula in these subjects that allow children with SLI to demonstrate some relative strengths.

The overall pattern of results indicates (1) that children with SLI are, as a group, at a disadvantage in core school subjects compared with non-language impaired peers, but (2) within these subjects, some children are attaining the levels expected of TD peers, and (3) there are, relative strengths in Science and, to a lesser extent, Mathematics. These findings have both theoretical and practical (pedagogical) implications; we consider both below.

### Relative performance in the three core subjects: theoretical implications

Comparisons across subject areas are not straightforward. The analyses are based on levels of attainment in the respective domains. It is controversial whether these can be regarded as equivalent (Coe 2008, Coe *et al*. 2008, Goldstein and Cresswell 1996, Newton 2012). It has been argued, for example, that to achieve the same grade in STEM subjects such as Mathematics and Science is harder than to achieve that grade in an arts subject, such as English (Coe *et al*. 2008). It is not the purpose of this paper to address this controversy but note that, on either perspective (subjects as equivalent, or Science as more difficult), the relative performance of children with SLI in different subject areas is of considerable interest to our understanding of the nature of the impairment and its ramifications.

It is reasonable to assume that impairments in language should impact on children's learning, in school subjects and elsewhere. This assumption is borne out in the present findings by the generally poorer Key Stage 2 outcomes for children with SLI. However, if language deficits affect learning differentially across school subjects, then questions arise concerning which particular aspects of language impairment have consequences beyond language itself and which particular aspects of school subjects allow (some) children with SLI to perform relatively well.

According to one influential theory of the cognitive associations of SLI, children with this condition have relatively spared declarative learning but impaired procedural learning (Ullman 2004, Ullman and Pierpoint 2005). Declarative learning includes ‘the learning, representation, and use of knowledge about facts (“semantic knowledge”) and events (“episodic knowledge”)’ (Ullman 2004: 235), while procedural learning encompasses ‘the learning of new, and the control of established, sensori-motor and cognitive “habits”, “skills”, and other [skilled] procedures’ (p. 237). Research has broadly supported Ullman's Procedural Deficit Hypothesis (PDH), with children with SLI showing impairments in procedural memory tasks but performance comparable to TD comparisons in declarative memory tasks (Lum *et al*. 2012).

While most school subjects are likely to incur demands on both modes of learning (Jones and Idol 2013), it could be that they vary in terms of the relative weighting they place on each. If so, then the present data suggest that English places greater demands on procedural learning capacities than do Science or Mathematics. This may seem counterintuitive—Science and Mathematics both involve acquiring new procedural skills (Baroody and Dowker 2013, Hiebert 2013, Scardamalia and Bereiter 2006)—but, importantly, the PDH predicts that the procedural deficit for children with SLI is fundamentally (though not uniquely, cf. Hsu and Bishop 2014) associated with language use. From a procedural perspective, language use entails processing inherently complex sets of rules governing the structural hierarchies and combinatorial possibilities of syntax, phonology, morphology and aspects of semantics (Ullman 2004); it follows that the effects of deficit(s) in procedural learning are most likely to be manifest, and most obdurate, in a highly linguistic domain, such as English.

Different school subjects may also vary in terms of the scope for compensatory adaptation (that is, using relative strengths in other capacities to compensate for deficits elsewhere; cf. Ullman 2004). Thus, much of school learning is likely to be impeded by verbal procedural deficits but, in some contexts, children with SLI may be able to draw on other relatively intact abilities. Declarative memory for visual information appears to be spared in SLI (Lum *et al*. 2012) and this may be a valuable compensatory ability in Science and some areas of Mathematics. Consistent with this view, Matson and Cline (2012) report that children with SLI did not differ from TD peers in performance in Science education tasks designed to minimize language requirements and to maximize opportunities to use visual and symbolic representations. Similarly, Donlan *et al*. (2007) found that children with SLI did not differ from TD peers on a test of arithmetic principles that required evaluation of symbolic expressions (in contrast to performance on other number tasks that involved greater amounts of spoken and written language). The present study is not able to contribute direct evidence of task-differentiated performance within each subject area, because our real-world measures (Key Stage 2 results) aggregate across many tasks. Nevertheless, our findings are consistent with the thesis that children with SLI are in many cases able to draw on other capacities to achieve satisfactory outcomes in some areas of school work, including Mathematics and Science.

### Relative performance in the three subjects: pedagogical implications

The first and foremost implication of findings of the different outcomes across different subject areas reported here is that they underscore that many children with SLI do have the potential to reach or exceed educational targets that are set at national levels for TD children (see also Conti-Ramsden *et al*. 2009, Dockrell *et al*. 2007, Johnson *et al*. 2010). Historically, the needs of many individuals with language impairments have been neglected or met inadequately, and educational and life prospects have been poor (Clegg *et al*. 2005, Johnson *et al*. 2010). Improving services are leading to improvements in attainment. For example, Conti-Ramsden *et al*. (2009) found that children with persisting SLI were obtaining one more qualification (GCSE) at the end of compulsory education in the 21st century than their counterparts were in the 1990s (Snowling *et al*. 2001). There is also evidence of individual differences in SLI. Not all children are at risk of poor educational attainment (Conti-Ramsden *et al*. 2009), thus, it is important to ensure that our expectations for children with SLI reflect the scope for positive outcomes.

The second implication is that we need to look carefully at the interaction of child characteristics and subject demands. If it is harder to achieve the equivalent grade in a STEM subject than an arts subject (Coe 2008), how are we to account for the relatively superior performance of children with SLI in the former compared with the latter? Part of the answer clearly lies in the difficulties that children with SLI have with language and hence, we infer, with arguably the most language-intensive core subject, English. But language is used, extensively, in Mathematics and Science, too, and is known to be a source of difficulties even to TD children. This suggests that another part of the answer is that some aspects of these subjects, at least as taught in the Key Stage 2 curricula, present opportunities to learn that are compatible with relative strengths in other abilities of children with SLI. For example, we have suggested above that these may include visual and symbolic representations that may be understood and manipulated with less reliance on verbal skills. A crucial practical implication is that educators working with children with SLI may want to assess such capacities, to consider whether increasing opportunities for visually based and symbolic learning is appropriate and to build upon these (see also Matson and Cline 2012). This is not to suggest that verbal aspects of Science or Mathematics education should (or can) be avoided, but that these may be better supported in contexts which engage relative strengths in other abilities (see Brigham *et al*. 2011 for a useful discussion of means of verbal support for learning disabled students in Science).

The third implication is that, regardless of whether subjects are formally included or not in national assessments, we need additional evidence concerning children with SLI's functioning in a greater range of subjects, and at other levels of the curriculum. Identifying potential relative strengths feeds into related ways of supporting young people with this condition, including career guidance and early work placements; areas in which individuals with SLI tend to be under-served at present (Beitchman and Brownlie 2013, Conti-Ramsden and Durkin 2012, Durkin *et al*. 2012).

## Conclusions

Children with SLI, as a group, are clearly at a disadvantage in core subject areas at the end of primary schooling, compared with national norms. However, the findings indicate both particular weaknesses and relative strengths. We found that English, the most language-dependent subject, is the one (of the three examined) in which children with SLI struggle most. No subject is language-free and performance in both Mathematics (teacher assessments) and Science (both teacher assessment and test results) is predicted to some extent by language ability. Nevertheless, some children with SLI are able to make considerable progress in these domains and it is possible that this is because there are aspects to each subject which draw on other capacities. Mathematics and Science can be relative strengths for children with SLI. Given the historical nature of our educational data, our findings are in need of replication with more recent cohorts of children and in other school systems, including, other countries). In addition, future research is needed which examines more closely which aspects of the Mathematics and Science curricula are least and most difficult for these children, and how their language-related needs in all subjects can be best met.

## References

[b1] Arvedson PJ (2002). Young children with specific language impairment and their numerical cognition. Journal of Speech Language and Hearing Research.

[b2] Baroody AJ, Dowker A (2013). The Development of Arithmetic Concepts and Skills: Constructive Adaptive Expertise.

[b3] Beitchman J, Brownlie EB (2013). Language Disorders in Children and Adolescents.

[b4] Bew P (2011). Independent Review of KS2 Testing, Assessment and Accountability.

[b5] Bishop DVM (1982). Test for Reception of Grammar.

[b8] Bishop DV, Adams C (1992). Comprehension problems in children with specific language impairment: literal and inferential meaning. Journal of Speech and Hearing Research.

[b9] Botting N, Faragher B, Simkin Z, Knox E, Conti-Ramsden G (2001). Predicting pathways of specific language impairment: what differentiates good and poor outcome. Journal of Child Psychology and Psychiatry.

[b10] Botting N, Simkin Z, Conti-Ramsden G (2006). Associated reading skills in children with a history of specific language impairment (SLI). Reading and Writing.

[b11] Brigham FJ, Scruggs TE, Mastropieri MA (2011). Science education and students with learning disabilities. Learning Disabilities Research and Practice.

[b12] Clegg J, Hollis C, Mawhood L, Rutter M (2005). Developmental language disorders—a follow-up in later adult life: cognitive, language and psychosocial outcomes. Journal of Child Psychology and Psychiatry.

[b13] Coe R (2008). Comparability of GCSE examinations in different subjects: an application of the Rasch model. Oxford Review of Education.

[b14] Coe R, Searle J, Barmby P, Jones K, Higgins S (2008). Relative Difficulty of Examinations in Different Subjects.

[b17] Conti-Ramsden G, Botting N (1999). Characteristics of children attending language units in England: a national study of 7-year-olds. International Journal of Language and Communication Disorders.

[b18] Conti-Ramsden G, Crutchley A, Botting N (1997). The extent to which psychometric tests differentiate subgroups of children with specific language impairment. Journal of Speech, Language, and Hearing Research.

[b19] Conti-Ramsden G, Durkin K (2012). Postschool educational and employment experiences of young people with specific language impairment. Language, Speech, and Hearing Services in Schools.

[b20] Conti-Ramsden G, Durkin K, Simkin Z, Knox E (2009). Specific language impairment and school outcomes. I: Identifying and explaining variability at the end of compulsory education. International Journal of Language and Communication Disorders.

[b21] Conti-Ramsden G, Knox E, Botting N, Simkin Z (2002). Educational placements and National Curriculum Key Stage 2 test outcomes of children with a history of SLI. British Journal of Special Education.

[b22] Conti-Ramsden G, ST Clair MC, Pickles A, Durkin K (2012). Developmental trajectories of verbal and nonverbal skills in individuals with a history of specific language impairment: from childhood to adolescence. Journal of Speech, Language and Hearing Research.

[b23] Cowan R, Donlan C, Newton EJ, Lloyd D (2005). Number skills and knowledge in children with specific language impairment. Journal of Educational Psychology.

[b24] Department For Education (DfE) (2000).

[b26] Dockrell J, Lindsay G, Palikara O, Cullen MA (2007). Raising the Achievements of Children and Young People with Specific Speech and Language Difficulties and Other Special Educational Needs Through School to Work and College.

[b27] Donlan C, Cowan R, Newton EJ, Lloyd D (2007). The role of language in mathematical development: evidence from children with specific language impairments. Cognition.

[b30] Durkin K, Fraser J, Conti-Ramsden G (2012). School-age prework experiences of young people with a history of specific languagee impairment. Journal of Special Education.

[b31] Durkin K, Mok PL, Conti-Ramsden G (2013). Severity of specific language impairment predicts delayed development in number skills. Frontiers in Psychology.

[b32] Durkin K, Shire B (1991). Language in Mathematical Education: Research and Practice.

[b33] Durkin K, Simkin Z, Knox E, Conti-Ramsden G (2009). Specific language impairment and school outcomes. II: Educational context, student satisfaction, and post-compulsory progress. International Journal of Language and Communication Disorders.

[b34] Ellerton NF, Clarkson PC, Bishop AJ, Clements MA, Keitel C, Kilpatrick J, Laborde C (1996). Language factors in mathematics teaching and learning. International Handbook of Mathematics Education Part 2.

[b35] Fazio BB (1999). Arithmetic calculation, short-term memory, and language performance in children with specific language impairment: a 5-year follow-up. Journal of Speech Language and Hearing Research.

[b36] Ginsburg HP, Barbarin OA, Wasik BH (2009). Early mathematics education and how to do it. Handbook of Child Development and Early Education: Research to Practice.

[b37] Goldstein H, Cresswell M (1996). The comparability of different subjects in public examinations: a theoretical and practical critique. Oxford Review of Education.

[b38] Harlaar N, Hayiou-Thomas ME, Dale PS, Plomin R (2008). Why do preschool language abilities correlate with later reading? A twin study. Journal of Speech, Language, and Hearing Research.

[b39] Hiebert J (2013). Conceptual and Procedural Knowledge: The Case of Mathematics.

[b40] Hsu HJ, Bishop DV (2014). Sequence-specific procedural learning deficits in children with specific language impairment. Developmental Science.

[b41] Johnson CJ, Beitchman JH, Brownlie EB (2010). Twenty-year follow-up of children with and without speech–language impairments: family, educational, occupational, and quality of life outcomes. American Journal of Speech–Language Pathology.

[b42] Jones BF, Idol L (2013). Dimensions of Thinking and Cognitive Instruction.

[b43] Justice L, Logan J, Kaderavek J, Schmitt MB, Tompkins V, Bartlett C (2013). Empirically based profiles of the early literacy skills of children with language impairment in early childhood special education. Journal of Learning Disabilities.

[b44] Kleemans T, Segers E, Verhoeven L (2011). Precursors to numeracy in kindergartners with specific language impairment. Research in Developmental Disabilities.

[b45] Kleemans T, Segers E, Verhoeven L (2012). Naming speed as a clinical marker in predicting basic calculation skills in children with specific language impairment. Research in Developmental Disabilities.

[b46] Lemke JL (1990). Talking Science: Language, Learning, and Values.

[b47] Lum JA, Conti-Ramsden G, Page D, Ullman MT (2012). Working, declarative and procedural memory in specific language impairment. Cortex.

[b48] MÄkinen L, Loukusa S, Laukkanen P, Leinonen E, Kunnari S (2014). Linguistic and pragmatic aspects of narration in Finnish typically developing children and children with specific language impairment. Clinical Linguistics and Phonetics.

[b49] Matson G, Cline T (2012). The impact of specific language impairment on performance in science and suggested implications for pedagogy. Child Language Teaching and Therapy.

[b50] Mercer N, Dawes L, Wegerif R, Sams C (2004). Reasoning as a scientist: ways of helping children to use language to learn science. British Educational Research Journal.

[b52] Miranda AE, Mccabe A, Bliss LS (1998). Jumping around and leaving things out: a profile of the narrative abilities of children with specific language impairment. Applied Psycholinguistics.

[b54] Negen J, Sarnecka BW (2012). Number-concept acquisition and general vocabulary development. Child Development.

[b55] Nelson KE, Welsh JA, Trup EMV, Greenberg MT (2011). Language delays of impoverished preschool children in relation to early academic and emotion recognition skills. First Language.

[b56] Newton PE (2012). Making sense of decades of debate on inter-subject comparability in England. Assessment in Education: Principles, Policy and Practice.

[b57] Norbury CF, Bishop DV (2002). Inferential processing and story recall in children with communication problems: a comparison of specific language impairment, pragmatic language impairment and high-functioning autism. International Journal of Language and Communication Disorders.

[b58] Raven JC (1986). Coloured Progressive Matrices.

[b60] Renfrew C (1991). The Bus Story: A Test of Continuous Speech.

[b61] Scardamalia M, Bereiter C, Sawyer K (2006). Knowledge building: theory, pedagogy, and technology. Cambridge Handbook of the Learning Sciences.

[b62] Semel E, Wiig E, Secord W (1987). Clinical Evaluation of Language Fundamentals—Revised.

[b63] Snowling M, Bishop DVM, Stothard SE (2000). Is preschool language impairment a risk factor for dyslexia in adolescence. Journal of Child Psychology and Psychiatry.

[b64] Snowling MJ, Adams JW, Bishop DVM, Stothard SE (2001). Educational attainments of school leavers with a preschool history of speech–language impairments. International Journal of Language and Communication Disorders.

[b65] Snowling MJ, Hulme C, Bailey AM, Stothard SE, Lindsay G (2011). Better Communication Research Project: Language and Literacy Attainment of Pupils During Early Years and Through KS2: Does Teacher Assessment at Five Provide a Valid Measure of Children's Current and Future Educational Attainments?.

[b66] Statacorp (2011). Stata Statistical Software: Release 12.0 [software].

[b67] Stobart G (2009). Determining validity in national curriculum assessments. Educational Research.

[b68] Strand S (2006). Comparing the predictive validity of reasoning tests and national end of Key Stage 2 tests: which tests are the ‘best. British Educational Research Journal.

[b69] Tippett C (2009). Argumentation: the language of science. Journal of Elementary Science Education.

[b71] Ullman MT (2004). Contributions of memory circuits to language: the declarative/procedural model. Cognition.

[b72] Ullman MT, Pierpont EI (2005). Specific language impairment is not specific to language: the procedural deficit hypothesis. Cortex.

[b73] Wechsler D (1992). Wechsler Intelligence Scale for Children—Third Edition—Revised UK.

[b74] Wellington J, Osborne J (2001). Language and Literacy in Science Education.

